# Coronary CT Angiography Incorporating Doppler-Guided Prospective ECG Gating in Patients with High Heart Rate: Comparison with Results of Traditional Prospective ECG Gating

**DOI:** 10.1371/journal.pone.0063096

**Published:** 2013-05-16

**Authors:** Min Li, Bing-bing Yu, Jian-hua Wu, Lin Xu, Gang Sun

**Affiliations:** 1 Department of Medical Imaging, Jinan Military General Hospital, Jinan, Shandong Province, China; 2 School of Bioscience and Bioengineering, South China University of Technology, Guangzhou, Guangdong Province, China; 3 Department of Medical Cardiology, Jinan Military General Hospital, Shandong Province, China; Centre Hospitalier Universitaire Vaudois, Switzerland

## Abstract

**Purpose:**

As Doppler ultrasound has been proven to be an effective tool to predict and compress the optimal pulsing windows, we evaluated the effective dose and diagnostic accuracy of coronary CT angiography (CTA) incorporating Doppler-guided prospective electrocardiograph (ECG) gating, which presets pulsing windows according to Doppler analysis, in patients with a heart rate >65 bpm.

**Materials and Methods:**

119 patients with a heart rate >65 bpm who were scheduled for invasive coronary angiography were prospectively studied, and patients were randomly divided into traditional prospective (n = 61) and Doppler-guided prospective (n = 58) ECG gating groups. The exposure window of traditional prospective ECG gating was set at 30%–80% of the cardiac cycle. For the Doppler group, the length of diastasis was analyzed by Doppler. For lengths greater than 90 ms, the pulsing window was preset during diastole (during 60%–80%); otherwise, the optimal pulsing intervals were moved from diastole to systole (during 30%–50%).

**Results:**

The mean heart rates of the traditional ECG and the Doppler-guided group during CT scanning were 75.0±7.7 bpm (range, 66–96 bpm) and 76.5±5.4 bpm (range: 66–105 bpm), respectively. The results indicated that whereas the image quality showed no significant difference between the traditional and Doppler groups (P = 0.42), the radiation dose of the Doppler group was significantly lower than that of the traditional group (5.2±3.4mSv vs. 9.3±4.5mSv, P<0.001). The sensitivities of CTA applying traditional and Doppler-guided prospective ECG gating to diagnose stenosis on a segment level were 95.5% and 94.3%, respectively; specificities 98.0% and 97.1%, respectively; positive predictive values 90.7% and 88.2%, respectively; negative predictive values 99.0% and 98.7%, respectively. There was no statistical difference in concordance between the traditional and Doppler groups (P = 0.22).

**Conclusion:**

Doppler-guided prospective ECG gating represents an improved method in patients with a high heart rate to reduce effective radiation doses, while maintaining high diagnostic accuracy.

## Introduction

With the clinical application of multi-detector CT scanning for imaging the coronary arteries, coronary CT angiography (CTA) has emerged as an attractive noninvasive diagnostic modality for detecting coronary artery disease [1]. However, since Einstein et al. estimated a significant number of potential radiation-induced neoplasms from coronary CTA [2], the high effective dose and potential adverse consequences of coronary CTA have aroused greater attention and have limited the general application of the technique [3].

Until now, many methods have been developed and applied to reduce the radiation dose, among which prospective electrocardiograph (ECG) gating is one effective method that has demonstrated the obvious advantage of allowing for a decreased patient dose [4]–[8]. However, it is only effective when the heart rate is low [9]. Traditionally, when the heart rate <65 bpm, diastole is recommended as the optimal pulsing window for a relatively longer phase with less motion of the coronary arteries. As the heart rate increases, however, the lengths of R-R intervals are shortened correspondingly, with diastole compressed significantly. As a result, it is difficult to produce an optimal image quality exclusively at diastole, as some patients should be scanned during systole [10]–[15]. In this case, the pulsing windows are usually expanded to cover both systole and diastole, which results in a higher radiation dose (1, 17, 18).

Numerous studies have mainly focused on the relationship between the heart rate and the optimal pulsing windows to guide the clinical utility of prospective ECG gating for patients with a high heart rate. The results, nevertheless, are contradictory, as the optimal pulsing window does not always change regularly with the heart rate. For example, Leschka et al. believed that the narrowest reconstruction window providing diagnostic image quality was 60–70% for a heart rate <60 bpm, 60–80% for a heart rate of 60–70 bpm, 55–80% for a heart rate of 70–80 bpm, and 30–80% for a heart rate >80 bpm (20), whereas Weustink et al. concluded the optimal exposure windows to be at 60–76%, 30–77%, and 31–47% for a heart rate ≤65 bpm, 66–79 bpm, and ≥80 bpm, respectively (19, 29). The possible reason may be that the physiological phase is associated not only with heart rate but also with other factors, such as filling pressures and heart function [16]. A β-blocker is therefore recommended to control the heart rate. Although using a β-blocker has been proven to be an effective approach with which to lower the patient's heart rate, it is ineffective and may have adverse effects in some patients [1].

A precise definition of an optimal ECG pulsing window is therefore required for prospective ECG gating scanning in patients with a high heart rate. Since Doppler ultrasound has been proven to be a helpful methodology with which to predict the physiological phase [17], it is hypothesized that, compared with the traditional prospective ECG-gating protocol, prospective ECG gating incorporating Doppler ultrasound analysis (Doppler-guided prospective ECG gating) has the potential to compress pulsing windows from both systole and diastole to systole or diastole in order to reduce patient dose [17]. In the present study, we evaluated the patient dose and diagnostic accuracy of CTA incorporating Doppler-guided prospective ECG gating in patients whose heart rate could not be brought under 65 bpm.

## Materials and Methods

### Study Population

Our study protocol and radiation dose measurements were approved by the Ethics Committee of Jinan Military General Hospital (Protocol S1). Written informed consents, including information about the risk of radiation and iodine allergic reactions, were obtained from all of the participants.

**Figure 1:**
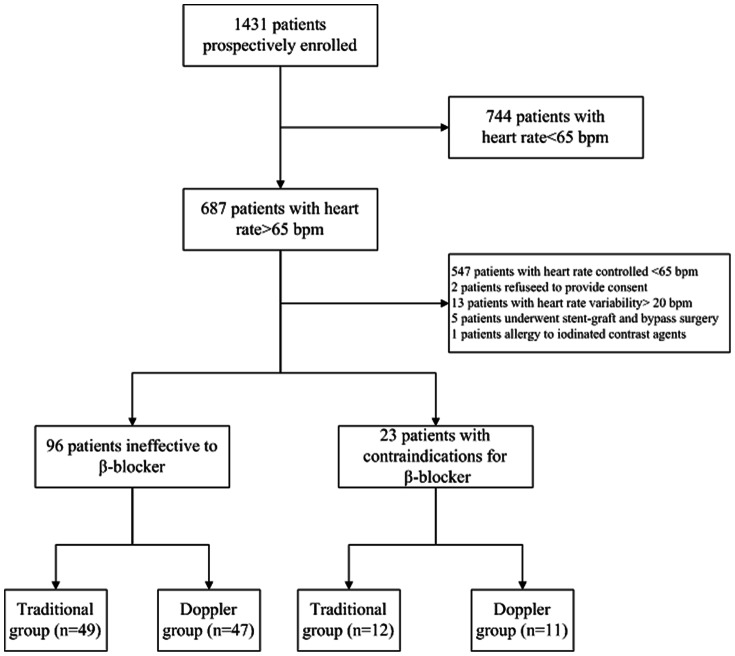
Flow Chart of References Searching. The diagram shows the exact criteria for the inclusion and exclusion of subjects.

In total, 1431 symptomatic patients who were scheduled for invasive coronary angiography (ICA) were prospectively studied. For patients with a heart rate >65 bpm, a β-blocker was traditionally applied in order to reduce the heart rate. Excluded from the study were 744 patients with a heart rate <65 bpm and 547 patients with a heart rate >65 bpm, whose heart rate could be brought under 65 bpm by the β-blocker. Among the 102 patients whose heart rate could not be brought under 65 bpm by the β-blocker as well as 38 patients with a heart rate >65 bpm and contraindications for β-blockers (asthma and severe chronic obstructive emphysema), the subjects were randomly divided into a traditional prospective ECG-gating group and a Doppler-guided prospective ECG-gating group. Patients who met the following criteria were excluded: refused to provide consent or withdrew for personal reasons (n = 2); demonstrated a heart rate variability of more than 20 bpm (n = 13); had undergone stent-graft and bypass surgery (n = 5); or were allergic to iodinated contrast agents (n = 1). Our final study cohort consisted of 119 patients, with 61 patients in the traditional prospective ECG-gating group and 58 in the Doppler-guided prospective ECG-gating group, respectively (Figure 1). No significant difference was found between the baseline characteristics of the two groups (traditional vs. Doppler) (Table 1).

**Table 1:** Baseline Characteristics of the Study.

Characteristics	Traditional prospective ECG gating	Doppler-guided prospective ECG gating	Total	P value
No. of patients	61	58	119	
Clinical feature				
Typical angina, n (%)	19 (31.2)	23 (39.6)	42 (35.3)	0.33
Atypical angina, n (%)	15 (24.6)	15 (25.9)	30 (25.2)	0.87
Abnormal ECG, n (%)	15 (24.6)	11 (19.0)	26 (21.9)	0.46
MI, n (%)	12 (19.7)	9 (15.5)	21 (17.6)	0.55
Demographics				
Age (y)	63.4±10.9	62.0±11.5	62.7±11.2	0.51
Male/female	42/19	43/15	85/34	0.52
BMI (kg/m^2^)	24.6±3.6	24.7±3.6	24.6±3.6	0.94
HR during scan (bpm)	75.0±7.7	76.5±9.0	75.7±8.4	0.32

Note: ECG = electrocardiogram; MI = myocardial infarction; BMI = body mass index, calculated as weight in kilograms divided by the square of height in meters; HR = heart rate.

### CT Angiography Protocol

All CT examinations were performed using 320-detector CT (Aquilion ONE, Toshiba, Nasu, Japan) with a detector collimation of 320×0.5 mm. For CT coronary imaging, a 40–60 ml bolus of Iohexol (Omnipaque 350 mg/ml; Amersham-GE Healthcare, Shanghai, China) was injected into an antecubital vein through an 18-gauge catheter at an injection rate of 4–6 ml/sec, followed by 50 ml of saline solution.

CT scanning parameters were set according to body mass index (BMI): 100 kV and 350 mA for BMI <18; 100 kV and 400 mA for BMI between 19–24; and 120 kV and 450–500 mA for BMI >24. For heart rates between 65–79 bpm, the two-heart-beat acquisition mode was used. For heart rates ≥80 bpm, the three-heart-beat acquisition mode was applied in order to increase the effective temporal resolution and obtain adequate imaging quality. Using the multi-heart-beat acquisition mode, the effective temporal resolution was able to reach 87.5–58.3 ms.

**Figure 2:**
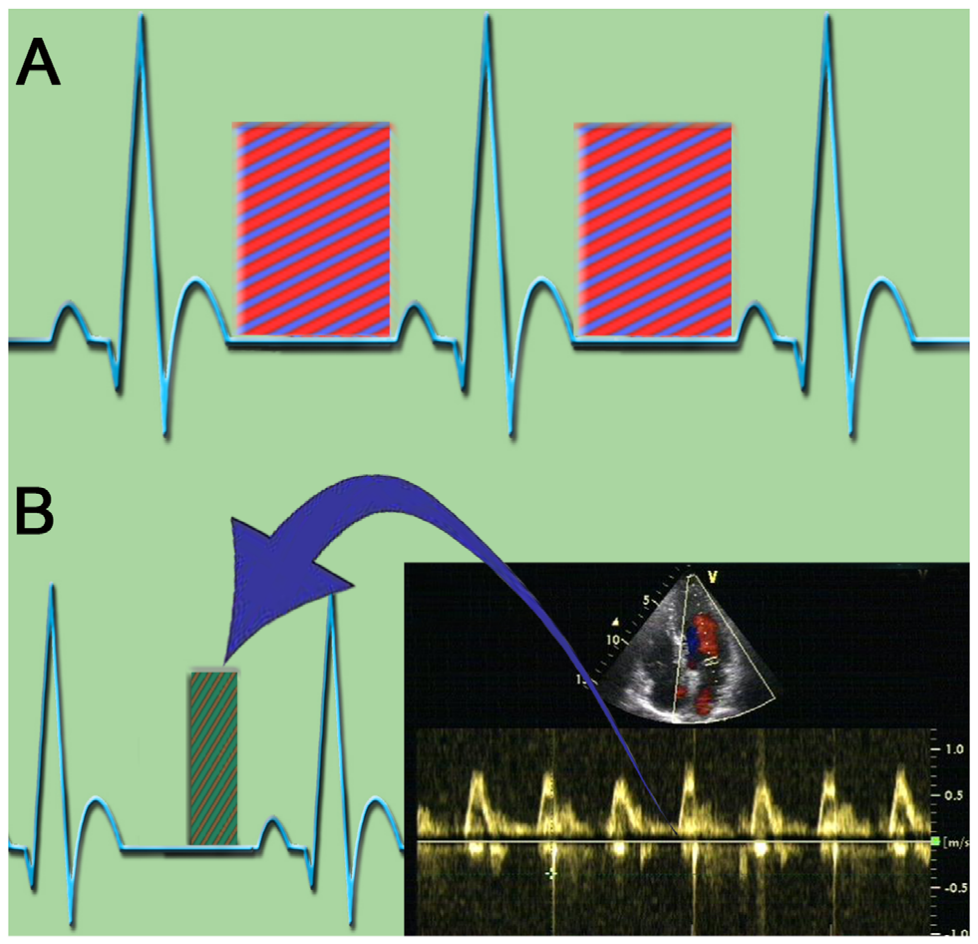
Methods of Traditional and Doppler-Guided Prospective ECG Gating. A) presents a schematic view of traditional prospective ECG gating. The exposure window is adjusted to cover both systole and diastole. B) offers a schematic view of Doppler-guided prospective ECG gating. The exposure window was adjusted at systole or diastole.

The exposure window of traditional prospective ECG gating was adjusted at 30%–80% of the cardiac cycle according to previous studies of the optimal pulsing windows of CTA_ENREF_19 (Figure 2A) [14], [15]. The pulsing intervals of Doppler-guided prospective ECG gating were determined by Doppler ultrasound analysis 5–10 minutes before CT angiography (Figure 2B). Transmitral pulsed-Doppler flow data were recorded from the transthoracic apical four-chamber using a clinical echocardiographic imaging system (Vivid 7; GE, USA), which was equipped within the same room with the CT. The patients were examined on the examining table of the CT scanner. The ECG data and Doppler data were recorded synchronously. The length of diastasis in diastole with less motion velocity was evaluated integrating the ECG signal. The end of the E-wave corresponded to the onset of diastasis (a), and the beginning of the late diastolic filling peak velocity (A-wave) corresponded to the end of diastasis (b) [18]. All data were recorded using the absolute timing (ms) from the previous R-peak. The length of diastasis was calculated by (b-a). When the length of diastasis was more than 90 ms, the pulsing windows were manually preset during diastole (during 60%–80%); if it was less than 90 ms, at which point the length was too short to reconstruct an image with good quality, the optimal pulsing intervals were moved from diastole to systole (during 30%–50%) [17].

### Reconstruction and Post Processing

Image reconstruction was performed with 0.5-mm slice thickness and 0.25-mm overlap. At the level of the middle right coronary artery (RCA), we used an absolute timing approach and reconstructed transverse images with a 10-ms step from the peak R-wave. When the interval with less motion artifact was determined, the whole-heart data were reconstructed. All reconstructed images were transferred to an independent workstation (Vitrea II FX, Vital Images, Minnetonka, MN, USA).

### Image Analysis

Coronary segments were defined by a 15-segment model according to American Heart Association guidelines [19]. All reconstructed images were evaluated and classified by two independent radiologists, who were blinded to the randomized protocol and ICA results. Image quality was classified into 4 grades: Grade 1—no artifacts and clear delineation of the segment; Grade 2—minor artifacts and mild blurring of the segment; Grade 3—moderate artifacts and moderate blurring; and Grade 4—severe artifacts and segment too poor for evaluation. The degree of involved lumen stenosis was measured using the narrowest dimension of the lumen at the level of stenosis compared with the normal lumen diameter distally. The measurements were made with electronic calipers in the Vitrea II FX workstation. The extent of vessel stenosis was classified into atherosclerosis with stenosis less than 50% and with stenosis greater than 50%. Consensus agreement was used for any disagreements.

### Invasive Coronary Angiography

Cardiac angiograms were performed using the conventional Judkin technique [20] within two weeks after CTA. Four views of the left coronary artery (LCA) and two views of the RCA were analyzed in consensus by two cardiologists. They were blinded to the CT results during analysis. Quantitative assessment of stenosis severity on angiograms was performed with the same criteria as those used for the CT data on the GE ADW4.2 workstation.

### Evaluation of Radiation Dose of CTA and ICA

The dose length product (DLP) displayed on the dose report of the CT scanner was recorded. An effective dose was obtained using the equation: E = k×DLP (k = 0.029 mSv×mGy^−1^×cm^−1^, which was calculated specifically for the 320-detector CT) [21]. For ICA, the effective dose was estimated as a product of the dose-area product (DAP) of the diagnostic coronary scenes × a conversion factor (k = 0.22 mSv/mGy×cm^2^) [22].

### Statistical Analysis

Statistical analysis was performed with the SPSS, version 16 software package for Windows (SPSS, Chicago, II, USA). A P-value of less than 0.05 was considered statistically significant.

Quantitative variables were expressed as mean ± SD, and categorical variables as frequencies or percentages. An inter-observer agreement for the determination of image quality was calculated with kappa statistics.

Results of ICA were used as the reference standard to calculate the sensitivity, specificity, positive predictive value (PPV), and negative predictive value (NPV) of CTA. A McNemar test was conducted to evaluate the difference between CTA and ICA results. The Fisher exact test and chi-square test, as appropriate, were used to evaluate the concordances for the groups based on the two types of prospective ECG gating (traditional vs. Doppler). In a sub-analysis, patients were subdivided into two groups according to mean heart rate (group A: 65 ≤ heart rate <80 bpm; group B: heart rate ≥80 bpm). Three-way ANOVA was performed to evaluate the radiation exposure, with groups (traditional vs. Doppler), heart rate (65 ≤ heart rate <80 bpm vs. heart rate ≥80 bpm), and imaging methodology (CTA vs. ICA) as factors.

## Results

The mean heart rate of the traditional-ECG gating group during CT scanning was 75.0±7.7 bpm (range, 66–96 bpm), with 72.4% (42/58) of a heart rate of 65 ≤ heart rate <80 bpm and 27.6% (16/58) of a heart rate ≥80 bpm. Results revealed a similar scanning heart rate for the Doppler ECG-gating group (76.5±5.4 bpm; range: 66–105 bpm, P = 0.32), with 80.3% (49/61) and 19.7% (12/61) in heart rates of the group for which 65 ≤ heart rate <80 bpm group and the groups for which heart rate ≥80 bpm, respectively. In the Doppler group, 15 patients were scanned during systole (30%–50%), and 43 patients were exposed during diastole (60%–80%).

### Image Quality

Of the 1,785 coronary arterial segments in 119 patients (15 segments per patient), 53 segments were excluded because the segments did not exist and could not be visualized by ICA and CTA. A total of 1,732 segments were thus available for analysis. The inter-observer agreement for image quality assessment was high (kappa = 0.78). The image quality grades of these segments were as follows: grade 1—81.9% (1419/1732); grade 2—12.9% (224/1732); grade 3—4.3% (74/1732); and grade 4—0.9% (9/1732). There was no significant difference in image quality score between traditional and Doppler-guided prospective ECG gating (P = 0.42) (Table 2).

**Table 2:** Image Quality in Different Sub-groups.

	Traditional prospective ECG gating		Doppler-guided prospective ECG gating	
	65–80 bpm	≥80 bpm	65–80 bpm	≥80 bpm
NO.	49	12	43	16
Heart rate (bpm)	71.8±4.0	87.7±5.4	71.9±4.1	88.5±7.3
Image quality				
Grade 1	85% (605/710)	72% (127/177)	82% (501/608)	78% (186/237)
Grade 2	11% (81/710)	20% (36/177)	13% (78/608)	12% (29/237)
Grade 3	3% (20/710)	7% (12/177)	4% (24/608)	8% (18/237)
Grade 4	1% (4/710)	1% (2/177)	1% (5/608)	2% (4/237)

### Effective Dose

**Figure 3:**
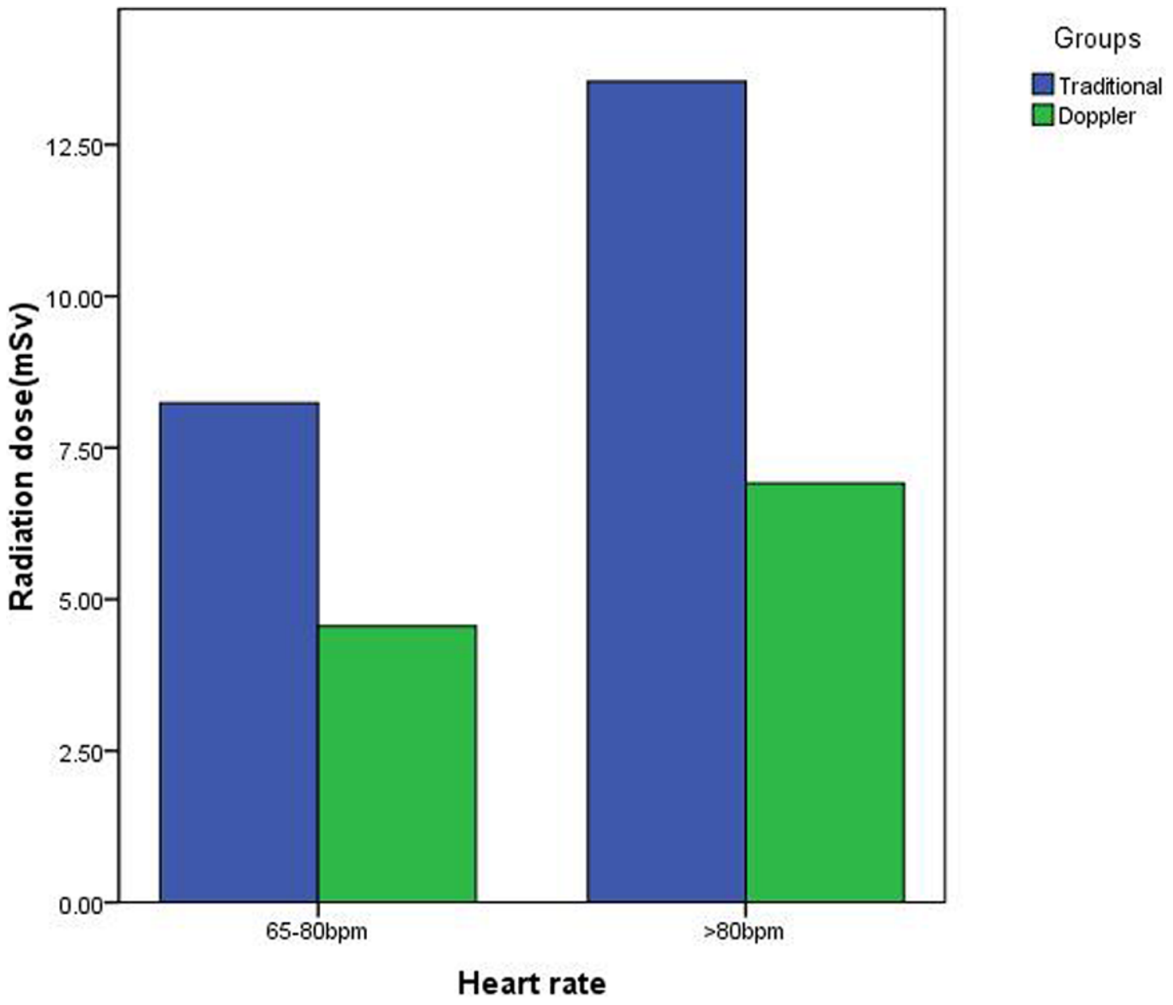
Dose of Traditional and Doppler-Guided Prospective ECG Gating. Overall, applying Doppler-guided prospective ECG gating, the patient dose of CTA was significantly lower than that of traditional prospective ECG gating.

Overall, the effective dose in the Doppler-guided prospective ECG-gating group was significantly lower than that in the traditional prospective ECG-gating group (F = 4.6, P = .003), and the patient dose in ICA was significantly higher than that of CTA (F = 123.1, P<0.001) (Figure 3). In addition, the radiation dose in patients with a heart rate ≥80 bpm was higher than that in patients with a heart rate between 65 bpm and 80 bpm (F = 15.7, P<0.001).

A groups × imaging methodology interaction followed by separate ANOVAs for traditional and Doppler-guided prospective ECG gating revealed that, whereas the effective dose of ICA was similar for the two groups (15.3±3.6 mSv vs. 15.0±5.2 mSv, respectively, P = 0.65), the radiation dose of CTA in the Doppler-guided prospective ECG-gating group (5.2±3.4 mSv) was lower than that of traditional prospective ECG gating (9.3±4.5 mSv, P = 0.001) (Figure 3). This interaction was further qualified by a groups × imaging methodology × heart rate interaction, which suggested that, whereas the dose of CTA in traditional group was lower than that of ICA at heart rates between 65 to 80 bpm (8.2±5.3mSv vs. 14.9±5.6mSv, P<0.001), the dose of CTA increased significantly in heart rates ≥80 bpm, with no significant difference from that of ICA (13.5±4.1 mSv vs. 15.1±3.5mSv, P = 0.1). In the Doppler group, on the other hand, the dose of CTA was lower than that of ICA in both the group with a heart rate of 65 ≤ heart rate <80 bpm (4.6±3.3mSv vs. 14.5±3.2mSv, P<0.001) and the group with a heart rate ≥80 bpm (6.9±3.2mSv vs. 17.4±4.1mSv, P<0.001).

### Diagnostic Accuracy

The inter-rater concordance between the two radiologists for ICA was good (kappa = 0.82). In the traditional prospective group, there were 154 segments in 44 patients with stenosis ≥50%. Among the 154 stenotic segments proven by ICA, CTA diagnosed 147 cases accurately. Among the 733 unstenotic segments proven by ICA, CTA diagnosed 718 cases accurately. The sensitivity of CTA was 95.5% (95% CI: 90.9, 97.8); the specificity was 98.0% (95% CI: 96.7, 98.8); the PPV was 90.7% (95% CI: 85.3, 94.3); and the NPV was 99.0% (95% CI: 98.0, 99.5), respectively. The McNemar test showed no significant difference in the diagnostic results between CTA and ICA (P = 0.13).

**Figure 4:**
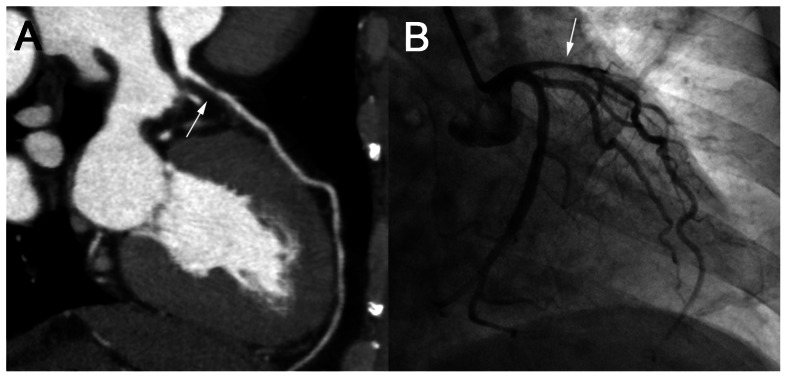
Example of CTA with Doppler-Guided Prospective ECG Gating. A male patient with a BMI of 21.5. The predicted length of DTD was 81 ms, and the exposure timing was preset at 30%–50% during the R-R interval. The scanning heart rate was 78 bpm. CTA with Doppler-guided prospective ECG gating (A) and ICA (B) showed stenosis of LAD (white arrows). The effective dose of CTA was 3.2 mSv.

In the Doppler-guided prospective ECG-gating group, 158 segments in 42 patients were diagnosed with stenosis ≥50% by ICA, among which 149 segments were detected by CTA. In the 687 segments with stenosis <50%, 667 segments were diagnosed accurately by CTA. For the 845 evaluated segments, the sensitivity of CTA was 94.3% (95% CI: 89.5, 97.0); the specificity was 97.1% (95% CI: 95.6, 98.2); the PPV was 88.2% (95% CI: 82.4, 92.2); and the NPV was 98.7% (95% CI: 97.5, 99.3), respectively. The McNemar test showed no significant difference in the diagnostic results between CTA and ICA (P = 0.06) (Figure 4). The diagnostic accuracies at patient and artery levels are presented in Table 3. In total, there was no statistical difference in concordance between the traditional and Doppler-guided prospective ECG-gating groups at patient, artery, and segment levels (P = 1.0, 0.48, and 0.22, respectively).

**Table 3:** Diagnostic Accuracy of Traditional and Doppler-Guided Group at Patient-, Artery- and Segment- levels.

	Sensitivity(95% CI)		Specificity(95% CI)		PPV(95% CI)		NPV(95% CI)	
	Traditional	Doppler	Traditional	Doppler	Traditional	Doppler	Traditional	Doppler
Patient level	97.0(84.7, 99.5)	96.8(83.8, 99.4)	89.3(72.8, 96.3)	88.9(71.9, 96.2)	91.4(77.6, 97.0)	90.1(76.4, 96.9)	96.2(81.1, 99.3)	96.0(80.5, 99.3)
Artery level	91.1(79.3, 96.5)	93.6(82.8, 97.8)	96.5(93.0, 98.3)	97.8(94.6, 99.2)	85.4(72.8, 92.8)	91.7(80.5, 96.7)	98.0(94.9, 99.2)	98.4(95.3, 99.4)
LM	100.0(20.7, 100.0)	100.0(34.2, 100.0)	100.0(94.0, 100.0)	100.0(93.6, 100.0)	100.0(20.7, 100.0)	100.0(34.2, 100.0)	100.0(94.0, 100.0)	100.0(93.6, 100.0)
LAD	93.8(71.7, 98.9)	92.7(68.5, 98.7)	95.6(85.2, 98.8)	97.7(88.2, 99.6)	88.2(65.7, 96.7)	92.9(68.5, 98.7)	97.7(88.2, 99.6)	97.7(88.2, 99.6)
LCX	92.3(66.7, 98.6)	88.9(67.2, 96.9)	93.9(83.5, 97.9)	95.0(83.5, 98.6)	80.0(54.8, 93.0)	88.9(67.2, 96.9)	97.9(88.9, 99.6)	95.0(83.5, 98.6)
RCA	86.7(62.1, 96.3)	100.0(77.2, 100.0)	95.7(85.5, 98.8)	97.8(88.4, 99.6)	86.7(62.1, 96.3)	92.9(68.5, 98.7)	95.7(85.5, 98.8)	100.0(92.0, 100.0)
Segment level	95.5(90.9, 97.8)	94.3(89.5, 97.0)	98.0(96.7, 98.8)	97.1(95.6, 98.2)	90.7(85.3, 94.3)	88.2(82.4, 92.2)	99.0 (98.0, 99.5)	98.7(97.5, 99.3)

Note: PPV = Positive predictive value; NPV = Negative predictive value.

## Discussion

Although prospective ECG gating has been suggested as a useful procedure to reduce radiation dose, it only works when the heart rate is low. A β-blocker is therefore recommended before CTA to control the heart rate, but is ineffective in some patients [9]. In this study, we aimed to evaluate the clinical performance of CTA incorporating Doppler-guided prospective ECG gating in patients with a high heart rate. The results indicated that compared with traditional prospective ECG gating, prospective ECG gating incorporating Doppler analysis could decrease radiation dose significantly while maintaining high diagnostic accuracy, especially for patients in whom the β-blocker proves ineffective and might have adverse effects.

Due to the consistent movement of the heart, it is critical to find a relatively tranquil duration in which to scan and reconstruct the coronary artery. Studies have confirmed that there are two durations suitable for image reconstruction. One corresponds to the diastasis in diastole, while the other takes place in systole [23]. Theoretically, when the heart rate ≤65 bpm, it is better to assess vessel lumen in diastole due to the increased blood flow, vasodilatation effect, and comparatively longer duration [13]. However, when the heart rate is increasing, the optimal pulsing windows fluctuate, rather than locating at diastole or systole stably or changing regularly with heart rate [10]–[12]. Thus, at a high heart rate, the optimal pulsing windows should cover both systole and diastole in order to maintain image quality, resulting in a significantly increased patient dose. Incorporating the results of previous studies, we selected 30%–80% as the pulsing windows for traditional prospective ECG gating [15].

The present study showed that Doppler-guided prospective ECG gating, which can analyze the true length of physiological phase, may offer the possibility of locating pulsing windows exclusively at systole or diastole and thus decrease radiation dose. Previous study has demonstrated that when the length of diastasis >90 ms, the optimal image quality could be obtained at diastole. Otherwise, when the length of diastasis <90 ms, the diastole is not suitable for optimal reconstruction, and the reconstruction windows should be set at systole [17]. As the pulsing windows were compressed from 30%–80% to 60%–80% or 30%–50%, the Doppler-guided prospective ECG gating could reduce radiation dose by nearly 44% of patient dose.

It is worth noting that in our study, the effective dose of Doppler-guided prospective ECG gating was still higher than those of previous studies based on patients with a low heart rate (5.2mSv vs. 2.2–4.2mSv) [1], [24], [25]. However, the present study showed a significant lower dose in patients with a high heart rate. In a study using 320-detector CT, when the heart rate >65 bpm, the radiation dose increased from 3.9 mSv in patients with a heart rate <65 bpm to 12.3 mSv, which was also two times more than the dose for Doppler-guided prospective ECG gating [1].

Generally, three main factors influence the radiation dose in patients with a high heart rate. The first is the length of exposure window. As mentioned above, this new technique of Doppler-guided prospective ECG gating cardiac CT angiography has the ability to compass the pulsing windows from 30%–80% to 60%–80% or 30%–50%. The second is the temporal resolution, determined by the hardware of CT scanners. If the temporal resolution is not sufficient to reconstruct a diagnostic image in patients with a high heart rate, the multi-segment reconstruction method should be applied to improve the effective temporal resolution, which increases the exposure time and patient dose significantly. Although the exposure windows were compressed greatly by Doppler-guided prospective ECG gating in the present study, the multi-segment reconstruction was applied by 320-detector CT, which led to a redundant dose when the heart rate is high [1]. As we can see from the results, the dose in patients with a heart rate >80 bpm was significantly higher than that in patients with a heart rate between 65 and 80 bpm for the application of the three-heart-beat acquisition mode. It is possible that dual-source CT (DSCT), with an advanced temporal resolution of 82.5 ms, could reduce the radiation dose further by applying Doppler-guided prospective ECG gating [26]. The third factor is the application of the iterative algorithm. The iterative reconstruction algorithm allows the dose to be lowered while maintaining image quality [27]. As the iterative algorithm was not applied in the first generation of 320-detectors CT, the radiation dose was still comparatively high. A recent study on the second-generation 320-detector CT scanner with iterative reconstruction in patients with a low heart rate showed a significantly reduced radiation dose, which could decrease radiation dose still further than the present CT scanner [28]. Therefore, the reduced radiation dose should not depend on one technique alone, but on a synthetic application of low dose techniques. Further study combining narrower pulsing windows, higher temporal resolution, and an iterative reconstruction algorithm may decrease the requisite radiation dose further.

Another reason for the redundant dose of the study was the differing conversion factor in dose estimation. Previous studies usually applied k = 0.014 mSv×mGy^−1^×cm^−1^ or k = 0.017 mSv×mGy^−1^×cm^−1^ as the conversion coefficient, which however is chest conversion factor. Recent studies have suggested that the conversion coefficient changes depending on organ, scanning mode, patient size, X-ray tube voltage, and other factors [29]. Applying dual-source CT, Hurwitz et al. suggested that the conversion factor for cardiac CT should be k = 0.025 mSv×mGy^−1^×cm^−1^ [30]. As mentioned above, Einstein et al. suggested that the conversion factor (k) should be 0.029 mSv×mGy^−1^×cm^−1^ for 320-detectors CT [21], which is significantly higher than the conversion factor used in previous studies.

It is worth noting that the procedure of CTA with Doppler-guided prospective ECG gating was more time-consuming and costly than the traditional method. However, patients whose heart rate could not be brought to desired levels only made up 8.3% of the total patients and thus did not disturb the work routine significantly. With regard to individual protection from radioactivity, Doppler-guided prospective ECG gating may reduce the patient dose by nearly 44%. Further studies should be conducted to investigate the cost-effectiveness of Doppler-guided prospective ECG gating in order to prove that the 44% of radiation dose saved benefits long-term survival.

There are two limitations to our study. First, patients with arrhythmia were excluded from this study, as it was difficult for Doppler to forecast the tranquil duration interval. Second, the study was performed by applying first-generation 320-detector CT. In the future, the advantages of Doppler-guided prospective ECG gating should be confirmed with other scanners.

In conclusion, in terms of decreasing the patient dose, ECG gating for coronary CTA improves from retrospective without dose modulation to retrospective with dose modulation to prospective mode. The current study indicates that Doppler-guided prospective ECG gating, a new method of ECG gating, may reduce effective doses further, while maintaining high diagnostic accuracy, particularly in patients with an elevated heart rate.

## Supporting Information

Protocol S1 **The Detailed Protocol Approved by the Ethics Committee of Jinan Military General Hospital.**
(DOC)
