# Supplementary material for: Coronary CT Angiography Incorporating Doppler-Guided Prospective ECG Gating in Patients with High Heart Rate: Comparison with Results of Traditional Prospective ECG Gating
Source: PLoS One. 2013 May 16;8(5):e63096. doi: 10.1371/journal.pone.0063096 (PMC3656032; doi:10.1371/journal.pone.0063096)
Supplement: Protocol S1 — The Detailed Protocol Approved by the Ethics Committee of Jinan Military General Hospital. (DOC) [file pone.0063096.s001.doc]

**Protocol S1 The Detailed Protocol Approved by the** **Ethics Committee of Jinan Military General Hospital**

With the clinical application of multi-detector CT scanning for imaging of the coronary arteries, CT coronary angiography (CTA) has emerged as an attractive noninvasive diagnostic modality for detecting coronary artery disease. However, since Einstein et al. estimated a significant number of potential radiation-induced neoplasms from coronary CTA, the high effective dose and potential adverse consequences of coronary CTA have aroused more attention and limited the general application of the technique. Generally, the effective dose (E) can be calculated by the equation: E = k × DLP, where k is the conversion factor. Mostly, the value of k was usually used as 0.017 mSv × mGy^−1^ × cm^−1^ . However, this value is initially used for radiation dose calculation in the chest. Hurwitz et al., using dual-source CT, proposed that the conversion factor should be 0.025 mSv × mGy^-1^ × cm^-1^ for cardiac CT scanning, while Einstein et al. , applying 320-detector CT, suggested the k value should be 0.029 mSv × mGy^−1^ × cm^−1^. Therefore, the [effective](http://www.iciba.com/effective/) [dose](http://www.iciba.com/dose/) of coronary CTA might have been even higher.

Until now, many methods have been developed and applied to reduce the required radiation dose. Prospective electrocardiograph (ECG) gating is one of the methods that have shown an obvious advantage in decreasing patient dose. Since CT data are acquired over only a fraction of the R-R interval, careful definition of an optimal ECG pulsing window is required in CT scanning.

Traditionally, the prospective ECG gating presets pulsing windows mainly depending on different heart rate groups. When heart rate is <65 bpm, diastole is recommended as the optimal pulsing window for a relatively longer diastolic phase . However, as heart rate increases, it is hard to predict the optimal reconstruction intervals accurately. In this case, the pulsing windows are suggested to be expanded to cover both systole and diastole, which results in a higher radiation dose . Though β-blocker has been proven to be an effective approach to control the patient’s heart rate, for some patients β-blocker is ineffective and may have adverse effects .

An approach to predict the optimal pulsing windows accurately is desired. Doppler has been proven a helpful methodology that can predict the optimal reconstruction windows before coronary CTA. It is hypothesized that, compared with the traditional-prospective ECG-gating protocol, which presets the pulsing window according to heart rate, prospective ECG gating incorporating Doppler analysis (Doppler-prospective ECG gating) has the potential to compress pulsing windows and reduce patient dose, especially for patients with a high heart rate . In the present study, we evaluated the patient dose and diagnostic accuracy of Doppler-prospective ECG gating in patients whose heart rate could not be controlled to under 65 beats per minute (bpm).

**Detailed Description:**

Generally, we will prospectively enroll at least 100 patients whose heart rate could not be controlled under 65bpm, who will be randomly divided to the traditional-prospective ECG-gating group or a Doppler-prospective ECG-gating group.

**Inclusion Criteria:**

1. Patients who are suspected of coronary artery disease
2. Patients were who were scheduled for invasive coronary angiography(ICA)
3. Signed written informed consent

**Exclusion Criteria:**

1. Refusal to provide consent or withdrawal for personal reasons
2. Heart rate variability more than 20 bpm before CT scanning
3. Underwent stent-graft and bypass surgery
4. Allergy to iodinated contrast agents
5. Heart rate<65bpm
6. Complex congenital heart disease
7. Evidence of active clinical instability or lifethreatening disease or inability to adhere to study procedures.
8. Age under 18
9. Renal insufficiency (creatinine level >120 µmol/L)
10. Pregnancy

**Detailed protocol**

1. If a patient is eligible for the study (see inclusion and exclusion criteria) and has given informed consent, he will undergo CT coronary angiography 3 or more days before ICA.
2. For coronary angiography, thereafter, randomization is performed to the traditional-prospective ECG-gating group and a Doppler-prospective ECG-gating group.
3. If the patient is randomized to Doppler-prospective ECG-gating strategy, the pulsing intervals of Doppler-prospective ECG gating were determined by Doppler analysis 5-10 minutes before CT angiography. Transmitral pulsed-Doppler flow data were recorded from the transthoracic apical four-chamber by an experienced sonographer using a clinical echocardiographic imaging system, which was equipped within the same room with the CT. The patients were examined on the examining table of the CT scanner. The ECG data and Doppler data were recorded synchronously. The length of diastosis in diastole with least motion velocity was evaluated integrating the ECG signal. When diastasis was more than 90ms, the pulsing windows were preset during diastole (during 60%-80%); if diastasis was less than 90ms, when the length was too short to reconstruct an image with good quality the optimal pulsing intervals were moved from diastole to systole (during 30%-50%). If the patient is randomized to traditional-prospective ECG-gating strategy, the exposure window of traditional-prospective ECG gating was adjusted to 30%-80% of the cardiac cycle.
4. For image quality analysis, the image quality was classified into 4 grades: Grade 1, no artifacts and clear delineation of the segment; Grade 2, minor artifa**ct**s and mild blurring of the segment; Grade 3, moderate artifa**ct**s and moderate blurring; Grade 4, severe artifacts and segment too poor for evaluation.
5. During stenosis analysis, the degree of involved lumen stenosis was measured using the narrowest dimension of the lumen at the level of stenosis compared with normal lumen diameter distally.
6. The dose length product (DLP) displayed on the dose report on the CT scanner was recorded. An effective dose was obtained using the equation: E = k × DLP ( k = 0.029 mSv × mGy^−1^ × cm^−1^, which was calculated specifically for 320-detector CT).
7. The image quality, diagnostic accuracy and patient dose of the two types of prospective ECG gating (traditional vs. Doppler) were compared.
